# Letter Addressed to the Respective Mayors of the Twelve Districts of Paris

**Published:** 1801-04

**Authors:** 


The following complete and satisfactory Account of the Progress of the Vaccine Inoculation in France, we have translated from the Moniteur (official Paper) of the 11th Ventose, which we received on the 7th of March.

### *Letter addressed to the respective Mayors of the Twelve Districts of Paris.*

Citizen Mayor,

THE Medical Committee established at Paris by the Society of Subscribers for the diffusion of the Vaccine Inoculation, knowing your desire to promote the public good, have the honour of proposing to you, under the auspices of Citizen Præfect of the Department of the Seine, to concur with him in the establishment and propagation of this new method of inoculation.—

It was first discovered in England by Dr. Jenner, and immediately prosecuted with the greatest success by the English physicians, and afterwards by many physicians of Germany, Geneva, and every state in Europe; this method has been only known in France for about one year, through the exertions of Citizen Larochefoucault Liancourt. In the last spring, Citizen Liancourt published a statement, in which he announced, in consequence of the observations of the English physicians, that the Vaccine is a disease much milder than the inoculated Small-pox; that it is not contagious like that, and nevertheless an effectual preservative against the Small-pox.

This announce attracted universal attention; but the more it promised, the more important it became to be assured that it could accomplish what it promised; this certainty could only be attained by experiments. To arrive at it, Citizen Liancourt opened a subscription, the produce of which is to be applied to obtain the experience necessary, to ascertain in France the truth of these assertions. A great number of citizens eagerly subscribed: The subscribers met, and named a Committee of administration to regulate the expences. The funds arising from the subscription have been hitherto sufficient, and an audited account will be laid before the public.

The Society have also named a medical committee, for the purpose of carrying on the experiments. This committee has devoted itself to the trust confided to it, and laboured with the most constant activity from its establishment to the present moment. It has, at least, inoculated more than a thousand, without one sinister event; nay, not one of those inoculated has been confined to his room a single day.

The

The inoculated had conftant intercourfe with thofe not inoculated, even fo intimate, as a mother, who had not had the Small-pox, fuckling her child under the difeafe without the leaft fymptom of contagion. This point appeared fo well eftablifhed, that the moft violent oppofers of the new method have not fuggefted the flighteft doubt on this head.

In a word, the vaccinated have been inceffantly expofed to the contagion of Small-pox, even by fleeping in the fame bed, and eating and drinking out of the fame veffels, without any effect. More than feventy-two have been inoculated for the Small-pox, yet none have taken the infection.

The Committee has thus, by numerous experiments, verified the obfervations of the Englifh phyficians; and is convinced of the truth of the three principal ftatements, viz.

1. The vaccine is a very flight difeafe.
2. It is not contagious.
3. It is an effectual prefervative againft the Small-pox.

The Committee is preparing a Report, in which, as well as rendering an account of its own labour, it will demonftrate thefe great truths, and eftablifh the public opinion with refpect to the MOST BRILLIANT AND MOST IMPORTANT DISCOVERY OF THE EIGHTEENTH CENTURY, TO WHICH FRANCE, EUROPE, AND THE WHOLE WORLD WILL BE INDEBTED FOR THE ANNIHILATION OF THAT MOST DESTRUCTIVE SCOURGE, WHICH HAS RAVAGED AND DESOLATED IT FOR SO MANY CENTURIES.

While expecting this grand Report, which its importance has occafioned to be delayed fo long, the Committee has given a fketch of it to Citizen Præfect, whofe diftinguifhed ardour for the diffufion of knowledge, the fciences, and arts, and love for every thing interefting to humanity, was well known to them.

They explained to him that more experiments were neceffary to render the Vaccine Inoculation perfect; that it was neceffary to inftruct and form the inoculators who are ftrangers to it; and that above all things, it was neceffary to extend this practice among the people, in order to render it familiar and general, and in fome degree to naturalize it throughout France, to the end that all might enjoy, and enjoy in the fulleft extent, fo great a benefit; that to attain fo defirable an end, the Committee muft have an eftablifhment in the centre of Paris, where both practitioners, as well as the leaft inftructed and pooreft of the people, whofe time is fo precious to them, might eafily and conveniently acquire, the one inftruction, and the other the neceffary gratuitous affiftance; that all may have it in their power, at their eafe, and when they judged proper, to come and fee, examine, clear up, affure themfelves, and finally determine termine from the appearance of the inoculated, all doing well, and the explanations of the inoculators, who would make them perceive, and even feel in fome degree the excellence of this method; that thefe were the only means by which inattention, careleffnefs, indolence, ignorance, malevolence, and every kind of prejudice, which always fet themfelves in oppofition to new eftablifhments, could be vanquifhed, particularly when the object is to render them popular.

The Citizen Præfect, who had the fame views, haftened to take fuitable meafures to form this ufeful eftablifhment, where the public, and above all, the indigent, might find the necef-fary fuccours.

The Committee did not reft here; their zeal for the com-forts of the poor, and efpecially for the extenfion of the art, made them defirous that an emanation from this central efta-blifhment might extend to all the twelve departments of Paris; and to you, Citizen Mayor, it belongs to promote this meafure in your department.

The Committee propofes to you to charge one of their mem-bers, whom they will appoint, to remove into a central part of your department, and to concert with you, with the members and the phyficians and furgeons of the Committees of Benevo-lence, and all other phyficians and furgeons whom you may judge proper to call in, on the moft effectual means of perfect-ing and extending the Vaccine Inoculation.

Affiftance will thus be found at hand by thofe who want it, and who, confequently, will become more numerous. The inoculators will be better inftructed; and that which is effential both to the interefts of humanity and the progrefs of the art, they will avoid the errors which are almoft inevitable, when the Vaccine Inoculation is practiced without inftruction and without experience. There is a fpurious vaccine, which per-fons unfkilled eafily confound with the genuine. This fpurious kind does not fecure the patient from the Small-pox. What danger then may be incurred by thofe who having only had this kind, and who believing themfelves by this operation fecured from the Small-pox, expofe themfelves to contagion without fear, and even with defign? And if the Small-pox happen to fupervene, the ignorant and malevolent never fail to attribute the event to the inefficacy of the *true Vaccine*; whereas they have only had a falfe one. For want of fufficient inftruc-tion, and more particularly of experience, we have our-felves been deceived. At the commencement of our labours, it happened to us to confound the falfe with the true; hap-pily, the habit which we have of obferving, and our extreme circumfpection, preferved us from the dreadful confquences which might have arifen from this error. Can one conftantly ftantly hope the fame refult from perfons lefs informed, lefs attentive, and above all, lefs exercifed in the very difficult art of making experiments? May even our error then be profitable to others, and preferve them from a fimilar one. The progrefs of the art requires, the interefts of humanity demand it.

Such, Citizen Mayor, are the motives of the propofitions which the Committee have the honour of laying before you; in accepting it, you will accomplifh their wifhes, and partake, Citizen Mayor, with the Præfect of the Department of the Seine, in the honour and the gratification of having concurred in preferving our fellow-citizens from the dreadful ravages of the Small-pox. Health and refpect.

Signed, on behalf of the Medical Committee,

GUILLOTIN.

